# Transition from Positive to Neutral in Mutation Fixation along with Continuing Rising Fitness in Thermal Adaptive Evolution

**DOI:** 10.1371/journal.pgen.1001164

**Published:** 2010-10-21

**Authors:** Toshihiko Kishimoto, Leo Iijima, Makoto Tatsumi, Naoaki Ono, Ayana Oyake, Tomomi Hashimoto, Moe Matsuo, Masato Okubo, Shingo Suzuki, Kotaro Mori, Akiko Kashiwagi, Chikara Furusawa, Bei-Wen Ying, Tetsuya Yomo

**Affiliations:** 1 Faculty of Science, Toho University, Funabashi, Chiba, Japan; 2 Graduate School of Frontier Biosciences, Osaka University, Suita, Osaka, Japan; 3 Graduate School of Information Science and Technology, Osaka University, Suita, Osaka, Japan; 4 Faculty of Agriculture and Life Science, Hirosaki University, Hirosaki, Aomori, Japan; 5 Exploratory Research for Advanced Technology (ERATO), Japan Science and Technology Agency (JST), Suita, Osaka, Japan; National Institute of Genetics, Japan

## Abstract

It remains to be determined experimentally whether increasing fitness is related to positive selection, while stationary fitness is related to neutral evolution. Long-term laboratory evolution in *Escherichia coli* was performed under conditions of thermal stress under defined laboratory conditions. The complete cell growth data showed common continuous fitness recovery to every 2°C or 4°C stepwise temperature upshift, finally resulting in an evolved *E. coli* strain with an improved upper temperature limit as high as 45.9°C after 523 days of serial transfer, equivalent to 7,560 generations, in minimal medium. Two-phase fitness dynamics, a rapid growth recovery phase followed by a gradual increasing growth phase, was clearly observed at diverse temperatures throughout the entire evolutionary process. Whole-genome sequence analysis revealed the transition from positive to neutral in mutation fixation, accompanied with a considerable escalation of spontaneous substitution rate in the late fitness recovery phase. It suggested that continually increasing fitness not always resulted in the reduction of genetic diversity due to the sequential takeovers by fit mutants, but caused the accumulation of a considerable number of mutations that facilitated the neutral evolution.

## Author Summary

The detailed results of a two-year *in vitro* thermal adaptive evolution experiment are described. A laboratory-evolved *E. coli* strain with an improved upper temperature limit, as high as 45.9°C, was acquired after 523 days of serial transfer, equivalent to 7,560 generations, in nutrient-limited medium. The complete daily records of cell growth exhibited universal two-phase fitness dynamics, a rapid growth recovery phase followed by a gradual increasing growth phase, throughout the entire evolutionary process. Genome-sequence analysis not only showed considerable escalation of the spontaneous substitution rate, but also revealed the transition from positive to nearly neutral in mutation fixation. Particularly, even with the rising fitness of bacterial cells, neutrality was observed in molecular evolution. These observations suggested that a discrete evolutionary mode occurred in the continuous evolutionary route, linking Darwinian adaptive selection with Kimura's neutral evolution. Such transition from beneficial to neutral path may be adopted as an evolutionary strategy robust to rigorous environmental changes.

## Introduction

Evolution experiments conducted in the laboratory allow direct temporal observation of the genetic and phenotypic alterations and the precise verification of evolutionary mechanisms [1]–[3]. As evolution usually triggers functional improvement in either biological activity or physiological fitness, positive selection may fix beneficial mutations within the population. On the other hand, most mutations in extant living organisms are often shown to be neutral to their fitness in studies of molecular phylogenetics [4] focusing on a specific target gene or protein. Combining Darwinian adaptive evolution and Kimura's neutral molecular evolution, it can be assumed that the observation of neutral mutations was because extant living organisms have been mostly in the fitness stationary phase, while the increasing fitness accompanied by beneficial mutations only occurred in a limited early period in the extensive timescale of evolution. This raised the question of whether the increasing fitness is related to positive selection, while the stationary fitness is related to neutral evolution. That is, whether there is any chance that a considerable number of non-beneficial mutations can be accumulated in an evolutionary period of continuously rising fitness.

To address this question, we designed an evolution experiment maintaining strong selection pressure to examine the temporal accumulation of mutations in molecular evolution. Living organisms generally survive within a limited range of temperature, and temperatures slightly higher than the upper limit of this range often lead to reduced growth fitness and may sometimes cause extinction. Evolution experiments with thermal selection are practical, as the environmental temperature can be precisely controlled in the laboratory.

As a classical model, the bacterium *Escherichia coli* has been investigated intensively with regard to its physiological responses to thermal stress. In contrast to thermal tolerance that temporarily rescues the cells from heat damage *via* activated production of heat shock proteins [5]–[7], thermal adaptation is the capacity to overcome thermal stress and maintain self-propagation at temperatures higher than the primary limitation, generally due to genetic and/or phenotypic changes taking place within cells over a relatively long period [8].

The mechanism of thermal adaptation has been poorly investigated because of a lack of suitable experimental models with newly acquired physiological properties. The successful development of thermal adaptive mutants grown at temperatures 0.8°C higher than the growth limitation of the ancestral clone [9] and the relevant evolutionary tradeoffs [10] highlighted the necessity of experimental approaches in the study of thermal adaptive evolution. Subsequent rigorous studies investigated the chromosomal changes [11], deletion and insertion of long fragments [12] and the relative heat-induced expression profiles [13], which may contribute to thermal adaptation. However, several essential issues remain unclear, including how high the upper temperature limit can be raised for *E. coli* propagation, how quickly the fitness (*e.g.*, growth rate) is improved during the evolutionary process and how many mutations occur over the whole genome, particularly how mutations are fixed in a population accompanying improvements in fitness.

To address how cells accumulate beneficial and/or neutral mutations in adapting to relatively high temperatures, we carried out thermal adaptive evolution of *E. coli* in the test tube, starting from an initial temperature of 36.9°C up to a final temperature of 44.8°C in increments of 2°C or 4°C. At every stepwise temperature shift, a rapid recovery of growth rate in the primary phase of approximately 250 – 300 generations and a subsequent gradually increasing phase appeared with the fitness increasing significantly throughout the entire 2-year serial transfer experiment. A thermally adapted strain with a 4.7°C improvement in the growth limit was acquired after 523 days, equivalent to 7,560 generations. Whole-genome sequence analysis of several selected cell populations in thermal evolution showed that the *Ka/Ks* ratio (non-synonymous mutation frequency over synonymous mutation frequency) switched from a high value to nearly unity. Intriguingly, the contribution of fixed mutations to the fitness turned from beneficial to neutral, while the growth rate rose continually. This transition was accompanied by the emergence of a mutator phenotype in the highly selective environment. Accordingly, we assumed that the extant organisms, improving their phenotypes gradually and continually, may have accumulated neutral mutations to maintain genetic diversity for forthcoming environmental changes.

## Results

### Greatly improved upper temperature limit of cell growth from thermal adaptive evolution

**Figure 1:**
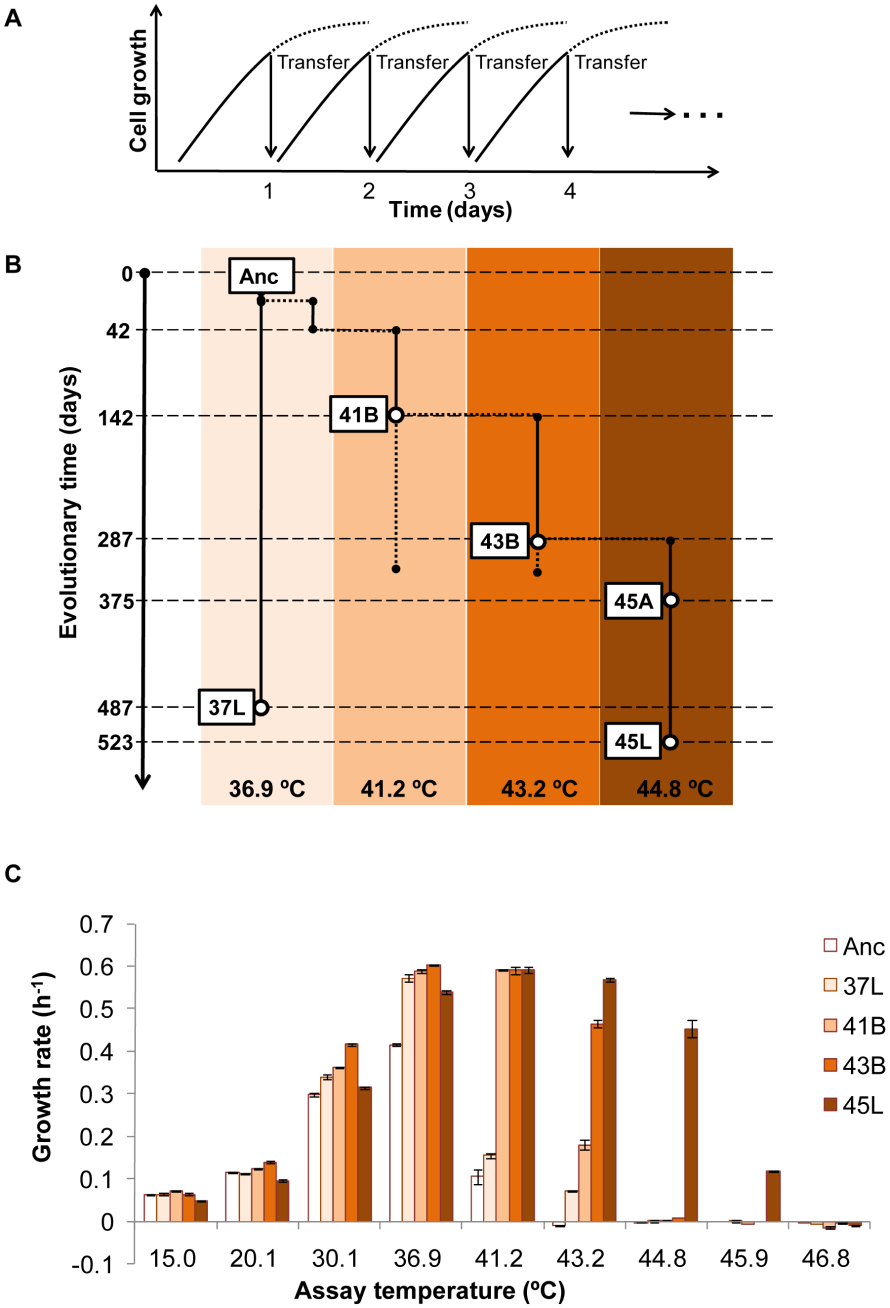
Thermal adaptive evolution. (A) Culture conditions in the evolution experiment. Daily serial transfer of cell culture was performed at the exponential growth phase. Daily growth curves are indicated by the solid and dotted lines, and the arrows indicate the serial transfer points. Serial transfer was continued for about 2 years. The vertical axis (cell growth) indicates the cell concentration on a logarithmic scale. (B) Scheme of the entire evolution experiment. The phylogeny and nomenclature of the experimental lineages evolved under defined laboratory conditions at different temperatures, indicated as 36.9°C, 41.2°C, 43.2°C and 44.8°C. Anc, 37L and 45L represent the ancestor, the 36.9°C and 44.8°C evolved strains, respectively. 41B and 43B indicate the strains at branching (temperature increase) points at 41.2°C and 43.2°C, respectively. 45A indicates an intermediate strain of the serial transfer at 44.8°C. The vertical axis represents the long-term evolution timescale. (C) Thermal growth characteristics of the bacterial strains acquired from the evolution experiment. The cell populations 41B and 43B, 45L, 37L and Anc, were evaluated. The averaged growth rates (± SE, *n* = 5–6) for each strain at 15.0°C, 20.1°C, 30.1°C, 36.9°C, 41.2°C, 43.2°C, 44.8°C, 45.9°C and 46.8°C are indicated. Constant propagation was defined as growth rate higher than 0.1 h^−1^.

Thermal adaptive evolution with a laboratory *E. coli* strain was carried out in a stepwise manner in increments of approximately 2°C or 4°C from 36.9°C to 44.8°C. Daily transfer of the culture in fresh medium was performed and the transfer point was tried to be kept during the exponential phase (Figure 1A). As shown in Figure 1B, the ancestor *E. coli* strain (Anc) with primary growth at 36.9°C (laboratory conditions) was divided into two: one for long-term culture at a constant temperature of 36.9°C (lineage of 37L) and the other (lineage of 41B-43B-45A-45L) for the thermal adaptive process with gradually increasing temperature of 36.9°C, 41.2°C, 43.2°C and 44.8°C. After 523 days of culture transfer, *E. coli* cells (designated as 45L) capable of rapid and constant growth at 44.8°C in minimal medium were obtained, as the endpoint thermal adaptive strain.

To see how much the evolution changed the growth profile of the *E. coli* cells, thermal niche assay was performed. Both Anc and 37L showed the highest growth fitness at 36.9°C but the restricted growth once the temperature was raised to 41.2°C, and ceased growth at the higher temperatures (Figure 1C). The evolved population (45L) continued to proliferate (>0.1 h^−1^) even when the temperature was raised as high as 45.9°C. A greatly extended range of growth temperature, a 4.7°C improvement in the upper limit, was finally achieved. Note that such a shift in the thermal niche was independent of the length of serial transfer but was directly related to the environmental changes, *i.e.*, the period of thermal stress (Figure S1). In addition, a slight trade-off due to the thermal adaptation was observed at the low temperatures of 15.0 and/or 20.1°C (Figure S2). We assumed that the thermal evolution experiment probably made the cells more sensitive to the drop in temperature.

### Two-phase fitness increasing dynamics in thermal evolution

**Figure 2:**
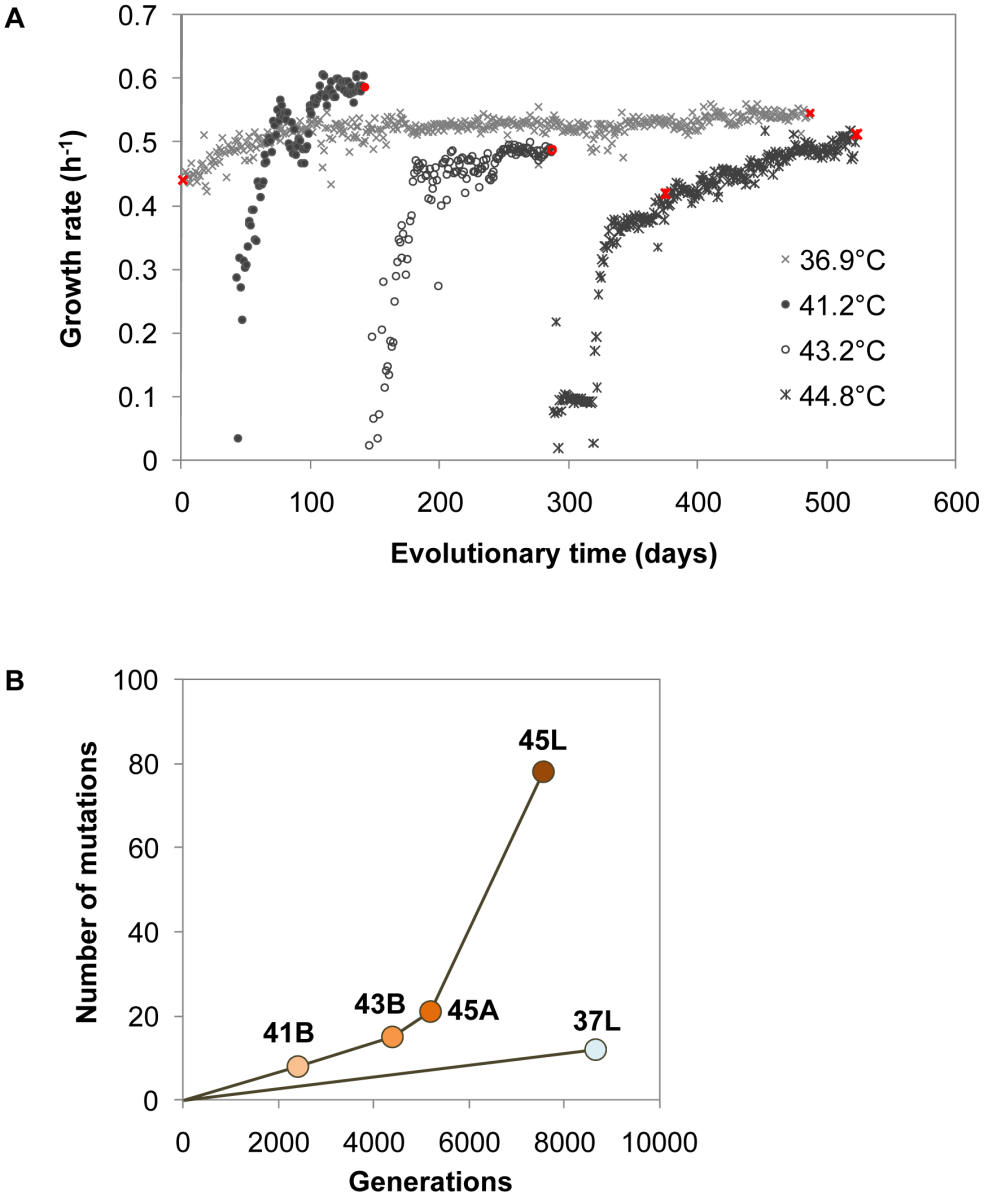
Fitness dynamics and genome mutations. (A) Trajectories of growth fitness during evolution. Daily cell growth rates at various temperatures were calculated according to the absorbance at 600 nm, as described in the Methods section. Grey crosses, closed circles, open circles and asterisks indicate the growth rates of the bacterial cells at 36.9°C, 41.2°C, 43.2°C and 44.8°C, respectively. The highlighted (in red) cell populations (Anc, 37L, 41B, 43B, 45A and 45L, as indicated in Figure 1B) were subjected to genome sequence analysis. (B) Accumulated genome mutations. Genome mutations occurred in the evolved bacterial populations were detected by array-based resequencing and/or the Sanger method. Anc, 37L, 41B, 43B and 45L were subjected to resequencing array and Sanger sequencing. Mutations in 45A were detected by Sanger sequencing according to the mutations that occurred in 45L. The numbers of total mutations (including single-nucleotide substitutions, insertions and deletions) in 37L, 41B, 43B, 45A and 45L were plotted against the generation of each population experienced from the ancestral clone (Anc).

The complete growth record revealed two periods involved in the adaptation process, *i.e.*, an initial rapid growth recovery phase followed by a gradual increasing growth phase (Figure 2A). Although the 2°C temperature shift resulted in a sudden growth rate drop (<0.1 h^−1^), the bacterial cells showed regular propagation (>0.3 h^−1^) within 40 days. Once the cells achieved a relatively high growth rate, a turning point appeared in the growth rate trajectory, where the subsequent period of gradual increase was initiated. This two-phase process was universally observed in all trajectories of diverse temperatures. In particular, the thermal evolution line showed continually and markedly increasing growth fitness, compared with the evolutionary route at a constant temperature of 36.9°C (lineage of 37L and refs. 2 and 14) [2], [14]. The trajectories observed here provided a detailed record of daily cell growth and clear insight into the dynamics of fitness recovery during thermal adaptation.

### Accumulation of genome mutations with continually rising fitness

The cell populations indicated in Figure 1B (marked in red, Figure 2A) were applied for mutation analysis. Genome mutation determination was based on a customised resequencing array technique and verified by Sanger sequencing (Table S3). In total, 12, 8, 15, 21 and 78 mutations (excluding mutations in rRNAs and tRNAs) were identified in 37L, 41B, 43B, 45A and 45L, respectively (Figure 2B and Table 1). The mutations occurring in the earlier period were inherited in later periods, *i.e.*, 43B included all 8 mutations that appeared in 41B, and 45L carried all mutations in 43B. The thermal evolution line accumulated mutations faster than the line under a constant temperature of 36.9°C, which may be attributed to the positive selection accompanied with the growth rate recovery during temperature adaptation. Intriguingly, the mutation fixation was markedly accelerated during the gradual recovery phase at 44.8°C (from 45A to 45L, Figure 2A), which indicated independence between mutation fixation rate and fitness recovery rate.

**Table 1:** Mutations occurring in the various evolution periods.

Evolutionary period	Number of SNPs	Number of InDels
Anc→37L	7	5
Anc→41B	6	2
41B→43B	3	4
43B→45A	2	4
45A→45L	46	11

The numbers of the single-nucleotide substitution (SNPs) and the small insertion and/or deletion (InDels) occurring in the various evolution periods were summarized. No large InDel was detected.

Both single-nucleotide substitutions (Table S1) and insertion/deletion mutations (Table S2) were found in all populations (Table 1). No large deletion neither prophage induction was observed, which was supposed to be thermal sensitive as reported in other bacteria [15]. Here we focused on the substitution analysis. Most mutations fixed during thermal evolution until 45A were nonsynonymous, which may result in changes in gene function. In contrast, from 45A to 45L, synonymous mutations were markedly accumulated, suggesting that the fixation rate of synonymous mutations increased even though they may have no significant contribution to the increase in fitness.

### Mutator appearance in the gradual growth recovery phase

The synonymous substitution rate was of the order of approximately 10^−10^ per bp per generation until 45A, whereas it was markedly increased to approximately 5.3×10^−9^ bp per generation from 45A to 45L (Table 2). As synonymous mutations are generally assumed to be close to neutral, the rate is proportional to the spontaneous synonymous substitution rate when the fixation mechanisms, genetic drift and/or hitchhiking effect, meet the condition that *Ne* × *u* (*Ne* and *u* are the effective population size and the spontaneous synonymous substitution rate, respectively) is sufficiently smaller than 1. When the initial population (*Ne,* less than 10^6^ cells in most daily transfers) and the general spontaneous mutation rate (*u,* 5.4×10^−10^ per nucleotide per generation [16]) are adopted, *Ne* × *u* is considerably smaller than 1. Thus, the observed acceleration of the synonymous substitution rate was most likely due to the increase in spontaneous substitution rate, which was subsequently verified by mutagenesis assay. It showed that the spontaneous substitution rate of 45L (∼1.0×10^−8^ per division) was approximately two orders higher than that of the ancestral clone (∼1.5×10^−10^ per division). The long-term thermal adaptive evolution resulted in a hypermutable phenotype.

**Table 2:** Synonymous substitution rate.

		Number of single-nucleotide substitutions						
Evolutionary period	Generation	Noncoding region	Synonymous (S)	Nonsynonymous (N)	*Ka* (10^−6^)	*Ks* (10^−6^)	*Ka/Ks*	Synonymous substitution rate (10^−10^)
Anc→37L	8659	0	1	6	1.9	1.0	1.8	1.2
Anc→41B	2405	0	1	5	1.6	1.0	1.5	4.3
41B→43B	1998	0	0	3	0.9	0.0	Infinite	0.0
43B→45A	788	0	0	2	0.6	0.0	Infinite	0.0
45A→45L	2369	2	12	32	10.0	12.5	0.8	53

*Ka* and *Ks* represent the frequencies of nonsynonymous (N) and synonymous (S) substitutions, respectively, with the unit of per nucleotide, and were calculated according to the following equations: 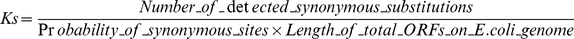,
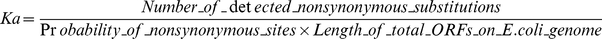

The probabilities of synonymous and nonsynonymous substitutions were 23.1% and 67.9%, respectively, based on the length of the ORF region of 4,148,273 bp (GenoBase, W3110, repeated sequences and overlapping regions among the ORFs were subtracted). Thus, the values applied here were 0.231 and 0.769 for the probabilities of synonymous and nonsynonymous sites, respectively, and 4,148,273 for the length of total ORFs in the *E. coli* genome. The synonymous substitution rate was *Ks* divided by the corresponding generation. The number of nonsynonymous substitutions from Anc to 43B, *i.e.*, 10, was significantly larger than the expected number based on the commonly observed synonymous substitution rate of 10^−10^ (see Discussion) and the above probabilities (one tailed tests, *P*<0.01).

Notably, mutations in the genes encoding enzymes with biological activities that may contribute to the accelerated spontaneous mutation rate were found in 45L (Table S1). For example, the single-nucleotide substitution producing a stop codon in *mutH* would cause protein translation to fail, resulting in loss of function of mismatch repair in DNA replication; the nonsynonymous substitution in *dnaE* replaced the nonpolar amino acid Ala with the polar amino acid Thr, which may cause structural changes in the protein leading to reduced fidelity of DNA polymerase III. The mutator was probably produced from these mutations related to replication fidelity or other related functions. This hypermutable property appeared not to affect the fitness improvement, but rather allowed the continually increasing growth rate (Figure 2 and Figure S3). The evolution experiment here showed not only the emergence of a mutator phenotype in the highly selective environment but also the important mutations potentially contributing to the high mutation rate.

### Transition from positive to nearly neutral mutation fixation in molecular evolution

The contribution of fixed substitutions to fitness, which was positive in the periods from Anc to 45A, turned to neutral or slightly negative in the period from 45A to 45L in terms of *Ka/Ks* ratio (Table 2). The biases for these probabilities (*Ks* and *Ka*) were calculated from the numbers of synonymous or nonsynonymous substitutions that occurred between the neighbouring populations of the intervening generation [17]. The high ratio of *Ka* to *Ks* (>1.0) in the periods from Anc to 45A suggested a positive selection effect during the evolutionary process [18]. The average contribution of each nonsynonymous substitution to the fitness increase (*2S*) was of the order of 1∼0.1 (Table 3), much larger than the threshold value (1/*Ne*, which was 10^−3^∼10^−6^ under our experimental conditions). It suggested that some or all of the nonsynonymous substitutions from Anc to 45A must have been fixed through positive selection.

**Table 3:** Contributions of nonsynonymous substitutions to fitness increase.

Period	Nonsynoymous (N)	Initial growth (h^−1^)	Final growth (h^−1^)	Relative fitness	Fitness increase (*2S*)
Anc→41B	5	0.27	0.59	2.19	0.17
41B→43B	3	0.02	0.49	24.50	1.90
43B→45A	2	0.08	0.42	5.25	1.29
Anc→45A	10	-	-	281.07	0.76

Initial growth and final growth are the growth rates on the first and last days during each period of diverse temperatures, respectively, as indicated in Table 2. Relative fitness indicates the ratio of the initial and final growth rates. Fitness increase (*2S*) represents the averaged increased growth fitness caused by each nonsynonymous substitution (N).

Despite the continually increasing growth rate in the period from 45A to 45L (Figure 2A and Figure S3), the *Ka/Ks* ratio was 0.8, suggesting that the majority of the fixed nonsynonymous substitutions were nearly neutral or slightly negative in their contribution to the fitness improvement. As the number of experimentally detected fixed nonsynonymous substitutions was 32, close to the theoretical value of 35 (calculated from the number of detected synonymous substitutions in Table 2), we assumed that the number of nonsynonymous substitutions responsible for the observed fitness improvement in the period from 45A to 45L was very small. These few positive substitutions were masked by the majority of nonsynonymous substitutions causing neutrality, leading to the nearly neutral value of the *Ka/Ks* ratio. Note that the *Ka* and *Ks* value acquired here agreed well with that analyzed using other methods considering the transversion and transition bias [19]. Taking into account the high fixation rate of synonymous mutations and the *Ka/Ks* ratio of 45A→45L, the average contribution of these fixed substitutions was approximately neutral even with the substantially increasing fitness.

## Discussion

We have shown that the average contribution of substitutions made a transition from positive to nearly neutral in the evolutionary process accompanied with continually increasing fitness that was defined as the growth rate. Although the cellular fitness showed common recovery in all the adaptation processes to the different temperatures, molecular evolution proceeded in two different manners: 1) mutation fixation of positive contribution to fitness, accompanied with fixation of few synonymous mutations; and 2) mutation fixation of nearly neutral contribution to fitness. As the spontaneous mutation rate increased significantly, both synonymous and nonsynonymous mutations were greatly accumulated and fixed. This large amount of mutations probably masked the positive contributions of a small number of mutations to fitness, leading to neutrality in terms of *Ka/Ks*.

The growth recovery constituted two phases universally throughout thermal evolution, which have been reported in other laboratory evolutionary experiments [3]. Beneficial mutations may occur in the early period resulting in rapid recovery of cell propagation, whereas the long-term response, the so-called “nearly static” state [14], [20], occurred in the later period for gradual continuing improvement of growth fitness. However, the growth recovery rate in the later period in thermal evolution was much higher than that supposed to be in the “nearly static” state. For example, the rate of fitness increase estimated by linear regression analysis was ∼10^−4^ per generation from 45A to 45L (Figure S3), and was ∼10^−5^ per generation in the period under a constant temperature of 36.9°C (from day 80 to 478 of the lineage 37L), which was consistent with that (∼10^−5^ per generation) in the period from 5K to 20K generation of the evolution carried out by Lenski's group [3]. These observations indicated that the elevated temperature triggered ∼10-fold acceleration of growth recovery in the gradual increasing phase.

Acceleration of the substitution rate has been predicted theoretically and investigated by experiments on mutators [21]–[25], although there have been few detailed reports regarding the precise genomic mutations that occur. We compare the mutation analysis of our evolutionary experiment with that of Lenski's group. Both Lenski's evolved clone 10K and the evolved 37L in this study that had experienced almost the same number of generations, showed approximately equivalent synonymous substitution rates of ∼1.0×10^−10^ per bp per generation (Table S4), although the two evolution experiments were performed independently in different laboratories with different *E. coli* strains under different growth selection pressures. The results agreed well with the general spontaneous substitution rate of 5.4×10^−10^ per bp per generation [16]. The endpoint clones, 40K (40,000 generations in ref. 3) [3] and 45L (this study, 7,560 generations) both showed similar mutation rates (Table S4), indicating that the mutator phenotypes appeared regardless of both selection pressure and the evolutionary process.

The transition from positive to near neutral of mutation fixation was mostly due to the accelerated spontaneous mutation rate. The *Ka/Ks* ratio was larger than unity from Anc to 45A, similar to that in the previous report (Table S4). Intriguingly, it was nearly unity from 45A to 45L, but was significantly larger than unity (*P*<0.01) from 20K to 40K in Lenski's experiment (Table S4). The fitness increase rate from 20K to 40K was expected to be not larger than to that from 10K to 20K, which was accompanied by neutrality of the fixed substitutions dependent on the specific classification of mutations [3]. The fitness increase rate from 45A to 45L was 10-fold higher accompanying nearly neutrality of fixed mutations regardless of the mutation category. Although difficulties in distinguishing between selective and neutral evolutionary processes have been noted [26], the study represents the first experimental verification of the positive to neutral transition of fixed mutation accompanying a continuous increase in growth fitness in a single evolutionary route. This result may have resulted from the temperature elevation before the cessation of fitness improvement in the evolution experiment.

The accelerated fixation rate of nonsynonymous mutation implied substantial genetic diversity in the population. The genetic diversity (*f*) of neutral substitution rate (*u*) show a difference at a single site based on the assumption of neutrality [27], [28]. The average number of nonsynonymous substitutions between the two cells over the whole genome is expected to be determined by the genetic diversity (*f*) and the number of all nonsynonymous sites in the genome. As *Ka/Ks* is nearly neutral from 45A to 45L, the synonymous substitution rate (*u*), 5.3×10^−9^ per base per generation, can be taken as the rate of neutral nonsynonymous substitution. When the population (*Ne*) is 10^6^ cells, the average number of nonsynonymous substitutions in the whole genome is approximately 3×10^4^. As this estimation of genetic diversity is based on the assumption that heterozygosity is in balance between spontaneous neutral nonsynonymous substitution and its loss by genetic drift in the population, the genetic diversity will decrease temporarily if specific mutants are amplified by positive selection or be overestimated if the population size is smaller than 10^6^. The high genetic diversity in the whole genome may have brought a burden from the negative contribution of nonsynonymous mutations to the population. However, the nearly neutral *Ka/Ks* value indicated that the burden was not so high. The mutation in *groEL* (Table S1), which appeared in the period from 43B to 45A, may have prompted the buffering performance that released misfolding and/or mutagenic stress [29], [31], or have modulated the codon adaptation [32] under the high temperature, thus allowing a large number of nonsynonymous mutations occurring after 45A to be nearly neutral. Additionally, the mutation was only detected in GroEL but not in other molecular chaperones strongly suggested the central role of chaperonin played in evolution.

The thermal adaptive process described here and the results of genome-wide mutation analysis showed that while cells increased their fitness in response to severe selection pressure, a substantial number of non-beneficial substitutions could be fixed. Thus, the neutrality observed in phylogenetic analysis on genes or proteins does not always mean that the fitness was in a stationary phase. Instead, in the changing environment, cells may accumulate nonsynonymous mutations to adopt the neutral path [33], [34] connecting multiple routes for adaptation to upcoming environmental changes [35], with accelerated nonsynonymous mutations as a robust survival strategy for sustainability. Further studies are required to determine the order of occurrence of all mutations and for quantitative evaluation of transcriptional alterations to investigate the molecular mechanism of the thermal adaptation in real-time. Evolution experiments in which gene mutations occur in various periods are not only valuable for investigating the genetic basis of adaptation and the relation between physiology and genetics, but are also valuable for molecular biologists to consider possible novel functions of these mutated genes or proteins involved in the evolutionary changes.

## Materials and Methods

### Bacterial strains

The bacterial strain *DH1ΔleuB::(gfpuv5-km^r^)* used for laboratory evolution was constructed from the wild-type *E. coli* strain *DH1* (National BioResource Project, National Institute of Genetics, Shizuoka, Japan), by replacing the chromosomal *leuB* with a foreign DNA fragment, PtetA-*gfpuv5-km^r^*, comprised of a reporter gene (*gfp*) and the kanamycin resistance gene (*km^r^*). The DNA fragment was amplified by PCR towards the plasmid, pGAG-2 [36], using the following primers: leuB-kanIG-f (5′-GCTCAACACAACGAAAACAACAAGGAAACCGTGTGATTAGAAA AACTCATCGAGCA-3′) and leuB-IGkan-r (5′-CGTCGAACAATTTTTCGTATAACGTCTTAGCC ATGAATTATCATTTGTAGAGCTCA-3′). Homologous recombination was performed as described previously [37], [38].

### Cell culture

Bacterial cells were cultured in 5 mL of modified M63 medium (62 mM K_2_HPO_4_, 39 mM KH_2_PO_4_, 15 mM ammonium sulfate, 1.8 mM FeSO_4_7H_2_O, 15 mM thiamine hydrochloride, 0.2 mM MgSO_4_7H_2_O, and 22 mM glucose) [38] supplemented with 2 mM leucine (Wako) and 25 µg/mL of kanamycin sulphate (Sigma) with shaking at 130 rpm. Cell culture was carried out at 36.9°C, 41.2°C, 43.2°C and 44.8°C using water bath shakers (EYELA NTS-4000A, EYELA NTS-4000E; Tokyo Rikakikai) and Personal-11 (Taitec). The water bath temperatures were measured using a Platinum Resistance Thermometer 5615 (Fluke). Serial transfer culture was performed by daily transfer of the cell culture with dilution in fresh medium prewarmed at 37°C. To avoid the pause of cell growth in the stationary phase, the serial transfer was tried to be carried out in the exponential phase. The dilution rate was theoretically determined to keep cell growth within log phase, *i.e.*, the cell concentration controlled as *OD*_600_ was 0.1–0.2 after 24 h in culture, according to the growth rate the day before. Such exponential growth occupied the majority of the evolutionary process. During the temperature upshift periods and the first 1–2 days of the culture restarted from the glycerol stock, the cell cultures were usually kept at the a relatively high concentration, *e.g.*, 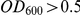. When the *OD*_600_ of the 24-h culture was below 0.1, another 24-h culture was performed instead of dilution or serial transfer. On the other hand, once the cell growth was paused, relatively higher *OD*_600_ value was adopted, for instance, during the periods at temperature rising point. This evolutionary culture process applied selection pressure on growth rate of *E. coli* cells. Note that during the laboratory evolution process, 5 cycles (10 days) of daily shuffling culture at 36.8°C and 43.2°C, and 2 cycles (4 days) at 36.8°C and 44.8°C were carried out due to the considerably slow growth after the temperature upshift from 41.2°C to 43.2°C and from 43.2°C to 44.8°C, respectively. Daily cell cultures were all stocked at –80°C. If the serial transfer culture was paused for any reason, it was restarted from the frozen stock. The growth rate was calculated according to the following formula: 
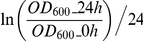
. Note that OD_600__0h was calculated in accordance with the *OD*_600__24*h* of the culture used for serial transfer and its dilution rate, *i.e.* 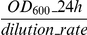. The dilution rate was determined by the growth rate the day before. Detail information on serial transfer and cell growth was summarized in Table S5.

### Thermal niche analysis

Anc, 37S, 37L, 41B, 41S, 43B, 43S, 45S and 45L cells were inoculated from the glycerol stock, and cultured at the corresponding adaptive temperature (36.8°C, 41.2°C, 43.2°C and 44.8°C, respectively). Following preculture at the described adaptive temperature for 24 h, all cell cultures were transferred to fresh medium and incubated at 15.0°C, 20.1°C, 30.1°C, 36.9°C, 41.2°C, 43.2°C, 44.8°C, 45.9°C or 46.8°C for 24 h. The thermal niche experiment was performed twice, and the growth rate at each culture temperature was calculated as the average value of a total of 5 or 6 replicates. Culture conditions, water bath shakers, thermometers and culture transfer (dilution) were applied as described in the “Cell culture” section.

### Mutagenesis assay

Mutation rate was determined according to previous reports [3], [39] with the following modifications. Cells were kept in the exponential growth phase under the same culture conditions as described in the “Cell culture” section. Cell concentration was counted by flow cytometry (FC500; Beckman) and verified by colony forming units (*cfu* assay). Approximately 1,000 cells (∼200 cells per mL) were inoculated into 5 mL of fresh mM63 medium supplemented with 2 mM leucine. Thirty cultures were used for each test. Cell culture was performed at the corresponding temperatures (36.9°C or 44.8°C) with shaking at 130 rpm for 20–28 h, and stopped once the cell concentration reached ∼1.0×10^7^ (for 45L) or ∼2.0 – 3.0×10^8^ (for Anc) cells per mL. The total cells in each tube were collected by centrifugation at 5,000 × *g* for 5 min and plated on agar mM63 plates with 2 mM leucine and 20 µg/mL of streptomycin. As the appropriate initial cell density on the agar plates (or inoculation in liquid medium) may vary with antibiotic concentration, we repeated the experiments with varied cell numbers for plating (10^8^ or 10^9^ cells per plate for Anc and 10^7^ or 10^8^ cells per plate for 45L). The plates were incubated at 37°C for 2 days. The numbers of plates without resistant colonies were counted, and the resultant probability was applied to estimate the spontaneous mutation rate.

### Genomic DNA preparation and microarray

The glycerol stocked cells were inoculated into mM63 medium supplied with 2 mM leucine, and grown until *OD*_600_ was approximately 0.5 with shaking at 130 rpm. The cell cultures were subsequently diluted to 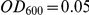
with fresh medium, and grown to stationary phase. Rifampicin (final concentration 300 µg/mL) was subsequently added and culture was continued for a further 3 h, to block initiation of DNA replication [40]. The cells were collected by centrifugation at 25°C at 16,000× *g* for 5 min, and the pelleted cells were stored at –80°C prior to use. Genomic DNAs were isolated and purified using a DNeasy Blood Tissue kit (Qiagen) for 37L and 45L, Aqua Pure Genomic DNA Isolation kit (Bio-Rad) for Anc, and Wizard Genomic DNA Purification kit (Promega) for 41B and 43B, in accordance with the respective manufacturer's instructions. Mutation detection by high-density oligonucleotide microarray analysis was performed using an Affymetrix GeneChip system. For preparation of genomic DNA, the assay procedures for use with the GeneChip *E. coli* Antisense Genome Array were carried out essentially according to the Affymetrix GeneChip Expression Analysis Technical Manual with slight modifications. To improve the efficiency of labelling, the incubation time was increased to 2 h.

### Design of genome-wide array-based resequencing

A fine-tuned resequencing array, covering the whole genome of *E. coli* W3110 strain (GenoBase, Japan, http://ecoli.naist.jp/GB6/search.jsp), was newly developed according to the Affymetrix CustomExpress Arrays [41]–[43]. Both strands of the genome were alternately tiled at single-base resolution using 21-mer perfectly matching (PM) probes. Furthermore, for each PM probe, we prepared three mismatching (MM) probes the centre bases of which (11^th^ base of the 21-mer oligo) were replaced by one of the possible three substitutions. Finally, a library consisting of a total of 18.6 million probes was arranged on three GeneChips for microarray analysis, covering four types of probe (1 PM and 3 MM) throughout the 4.6-Mb *E. coli* genome. The accuracy of the developed high-density oligonucleotide array is close to other next generation resequencing technologies (Ono *et al*., manuscript in preparation).

### Mutation detection

Mutations were identified by detecting differences in probe intensity between the ancestral (Anc) and thermal adaptive strains (41B, 43B and 45L). Single-nucleotide substitutions were identified by both loss of signal (reduced intensity of probes covering the substituted base) and gain of signal (increased intensity of any MM probes the centre bases of which matched the substitution). When the intensity of a number of neighbouring probes showed a significant decrease without any gain signals from MM probes, the region covered by these probes was considered a deletion. The deleted regions (Table S2) were excluded from the mutation rate calculation. In addition, as microarray-based resequencing is unreliable for the detection of either transposition or repeated sequences (multiple copies), we neglected the signals in the regions of rRNA, tRNA and insertions.

The precise single substitutions were determined by maximum likelihood estimation. Probe intensity was given as *I(i, b)*, where *i* and *b* represent the position on the genome and the nucleotide type of the central base in the probe, respectively. The average of the squared error between two strains was calculated by the following equation (1):
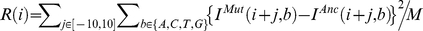
(1)


where *I^Mut^* and *I^Anc^* denote the intensity of mutant and ancestor, respectively, and *M* is the number of probes in the averaged window. Followed by filtering the candidate regions {Λ*_l_*} of mutation where R(Λ*_l_*) >τ, we evaluated the logarithmic likelihood ratio L(*i*) of all possible positions and the types of substitution within the range of each candidate region Λ*_l_*. L(*i*) was defined by the average of the squared error between the expected and observed intensity ratios. The expected intensity was computed as a function of the hybridisation free energy estimated according to the nearest neighbour model [41], [44] modified by introducing the mismatch effect to predict the intensity of mismatched probes. The position and base type of the substitution were identified (*P*<10^−4^) and re-evaluated using the likelihood ratio test.

## Supporting Information

Figure S1 Schematic drawing of evolution experiment and the thermal profile. Phylogeny and nomenclature (A) of experimental lineages evolved under defined laboratory conditions at different temperatures indicated as 36.9°C, 41.2°C, 43.2°C and 44.8°C. Anc, 37L, 41B, 43B, 45A and 45L are described in Figure 1B. 37S, 41S, 43S and 45S indicate the cell populations passaged at 333 days in culture from the ancestor. The thermal profiles of these bacterial cells were exposed to different temperatures but the same period of 333 days for culture transfer was determined (B). The average growth rates (± SE, n = 6) for each strain at 15.0°C, 20.1°C, 30.1°C, 36.9°C, 41.2°C, 43.2°C, 44.8°C and 45.9°C are indicated. The *E. coli* cells exposed to 333 days of laboratory evolution showed thermal profiles similar to that shown in Figure 1C.
(0.23 MB TIF)

Figure S2 Growth rate as a function of temperature. The growth rate of the cell populations 41B and 43B, 45L, 37L and Anc were evaluated as shown in Figure 1C. The averaged growth rates (n = 5–6) for each strain at 15.0°C, 20.1°C, 30.1°C, 36.9°C, 41.2°C, 43.2°C, 44.8°C, 45.9°C and 46.8°C are indicated. The logarithm of growth rate (h^−1^) is plotted against the inverse of the absolute temperature (° K). The inset represents the enlarged view of the linear range from 15.0°C to 36.9°C.
(0.17 MB TIF)

Figure S3 Fitness increase in the late period of the two-phase growth recovery dynamics. Daily growth rate (A) and fitness increase rate (B) of two lineages evolved at 36.9° C (37L) and 44.8° C (45L) are plotted. The upper and lower panels show the growth trajectories from day 80 to 487 (at 36.9° C) and from day 375 to 523 (at 44.8° C), representing the late periods of Anc80 to 37L and 45A to 45L, respectively. The generations were counted from day 80 (Anc80) and 375 (45A), respectively. Linear fitting of the growth fitness increase was indicated as the solid lines. The slopes are approximately ∼10^−6^ (A, upper), ∼10^−5^ (A, bottom), ∼10^−5^ (B, upper) and ∼10^−5^(B, bottom), respectively, indicating one-order difference in fitness increase between the two evolutionary lineages.
(0.32 MB TIF)

Table S1 Single-nucleotide substitutions occurred in the various evolution periods. The names (Genes), IDs (JWIDs), genome positions and functions of the genes or noncoding regions (Not ORF) are from GenoBase, Japan (http://ecoli.naist.jp/GB6/search.jsp), based on the genome information of W3110. N and S indicate synonymous and nonsynonymous substitutions, respectively. The noncoding regions are indicated by the neighbour genes.
(0.05 MB XLS)

Table S2 Insertions and deletions. The insertions and/or deletions appearing in each evolution period were detected by array-based resequencing and confirmed or determined by Sanger sequencing. The names (Genes), IDs (JWIDs) and genome positions of the genes or noncoding regions (Not ORF) are from GenoBase, as described in Table S1.
(0.03 MB XLS)

Table S3 Primers for Sanger sequencing. The primers used to confirm or identify the mutations involved in each gene or noncoding region are shown.
(0.05 MB XLS)

Table S4 Synonymous substitution rates from two evolution experiments. The numbers of single-nucleotide substitutions and the generations of Lenski's (asterisks) evolved bacterial cells were obtained from Barrick et al [3]. The analyses were performed as described in Table 2.
(0.03 MB XLS)

Table S5 Record of daily serial transfer.
(0.18 MB XLS)
