# Supplementary figures and images for: Transition from Positive to Neutral in Mutation Fixation along with Continuing Rising Fitness in Thermal Adaptive Evolution
Source: PLoS Genet. 2010 Oct 21;6(10):e1001164. doi: 10.1371/journal.pgen.1001164 (PMC2958811; doi:10.1371/journal.pgen.1001164)

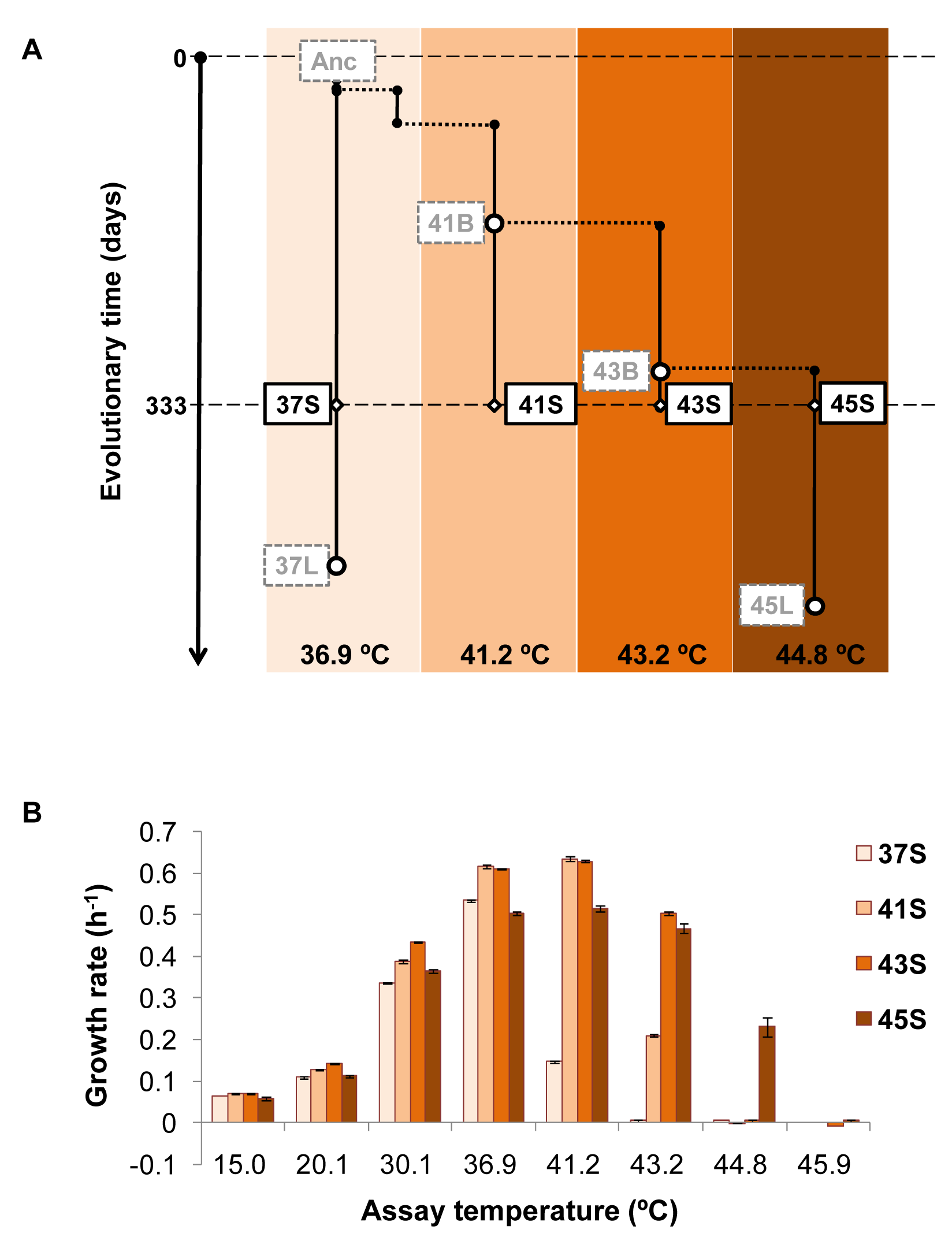

Supplement: Figure S1 — Schematic drawing of evolution experiment and the thermal profile. Phylogeny and nomenclature (A) of experimental lineages evolved under defined laboratory conditions at different temperatures indicated as 36.9°C, 41.2°C, 43.2°C and 44.8°C. Anc, 37L, 41B, 43B, 45A and 45L are described in Figure 1B. 37S, 41S, 43S and 45S indicate the cell populations passaged at 333 days in culture from the ancestor. The thermal profiles of these bacterial cells were exposed to different temperatures but the same period of 333 days for culture transfer was determined (B). The average growth rates (± SE, n = 6) for each strain at 15.0°C, 20.1°C, 30.1°C, 36.9°C, 41.2°C, 43.2°C, 44.8°C and 45.9°C are indicated. The E. coli cells exposed to 333 days of laboratory evolution showed thermal profiles similar to that shown in Figure 1C. (0.23 MB TIF) [file pgen.1001164.s001.tif]

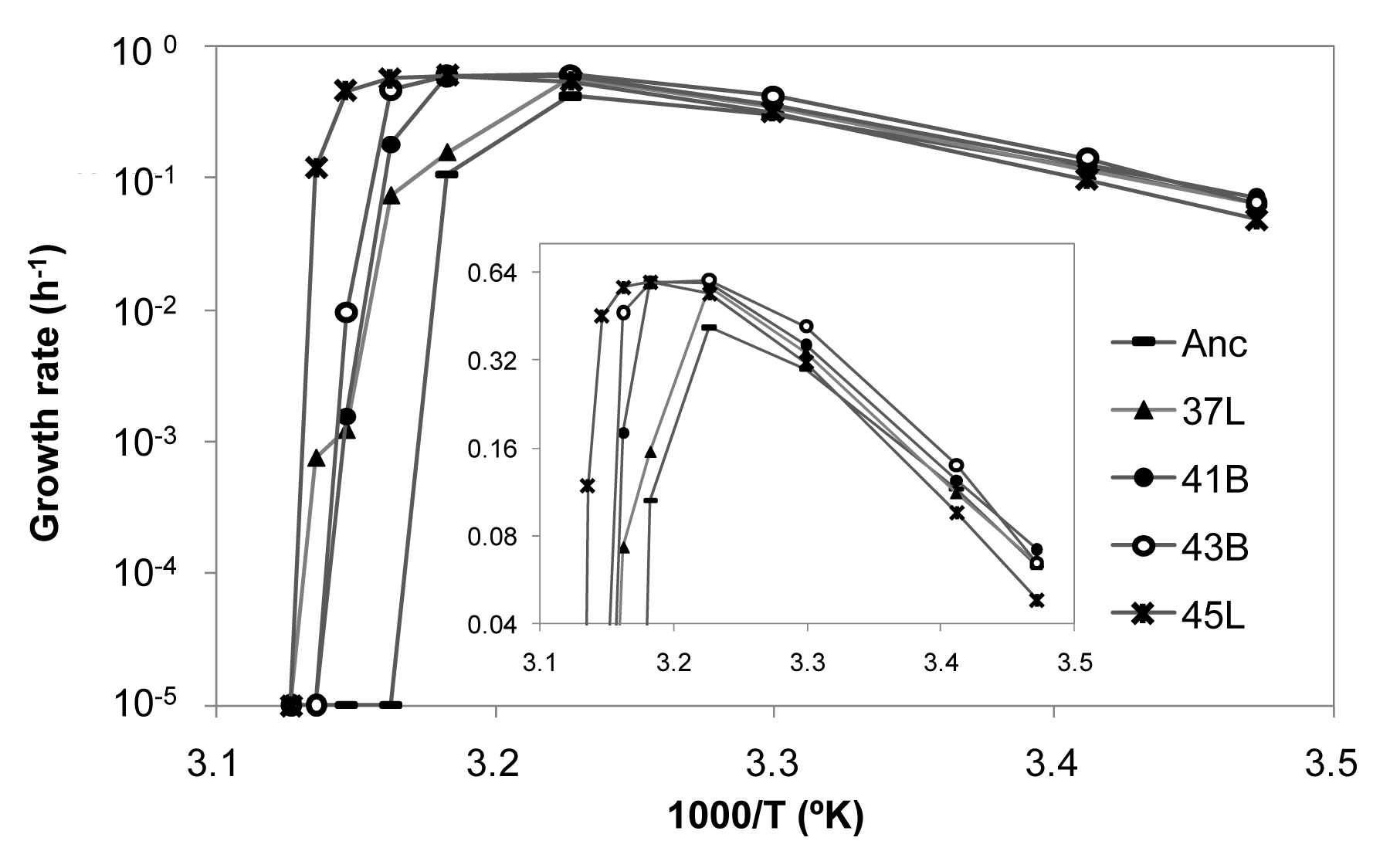

Supplement: Figure S2 — Growth rate as a function of temperature. The growth rate of the cell populations 41B and 43B, 45L, 37L and Anc were evaluated as shown in Figure 1C. The averaged growth rates (n = 5–6) for each strain at 15.0°C, 20.1°C, 30.1°C, 36.9°C, 41.2°C, 43.2°C, 44.8°C, 45.9°C and 46.8°C are indicated. The logarithm of growth rate (h−1) is plotted against the inverse of the absolute temperature (° K). The inset represents the enlarged view of the linear range from 15.0°C to 36.9°C. (0.17 MB TIF) [file pgen.1001164.s002.tif]

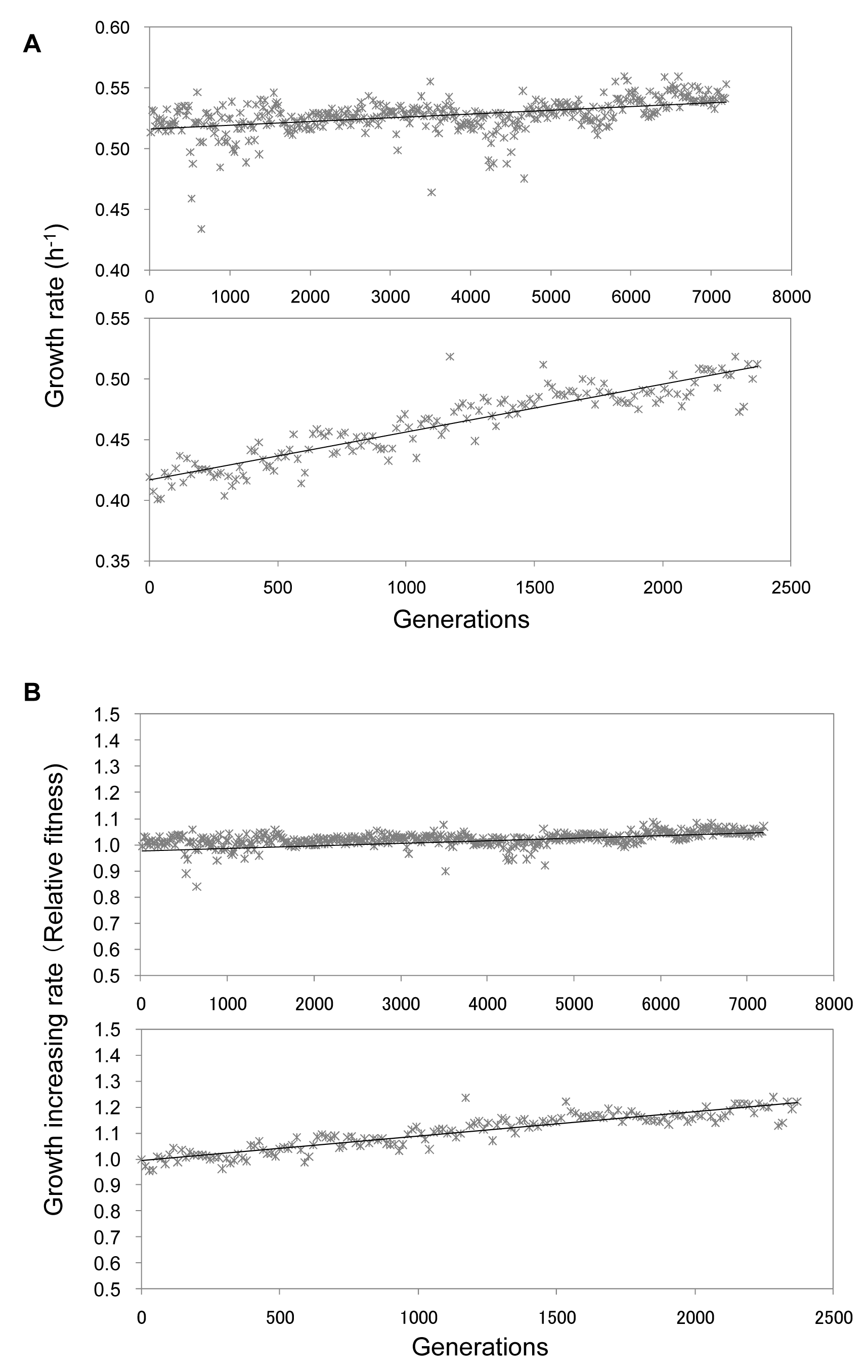

Supplement: Figure S3 — Fitness increase in the late period of the two-phase growth recovery dynamics. Daily growth rate (A) and fitness increase rate (B) of two lineages evolved at 36.9° C (37L) and 44.8° C (45L) are plotted. The upper and lower panels show the growth trajectories from day 80 to 487 (at 36.9° C) and from day 375 to 523 (at 44.8° C), representing the late periods of Anc80 to 37L and 45A to 45L, respectively. The generations were counted from day 80 (Anc80) and 375 (45A), respectively. Linear fitting of the growth fitness increase was indicated as the solid lines. The slopes are approximately ∼10−6 (A, upper), ∼10−5 (A, bottom), ∼10−5 (B, upper) and ∼10−5(B, bottom), respectively, indicating one-order difference in fitness increase between the two evolutionary lineages. (0.32 MB TIF) [file pgen.1001164.s003.tif]
